# Examining the Role of Race/Ethnicity and Sex in Modifying the Association Between Early Smoking Initiation and Mortality: A 20-Year NHANES Analysis

**DOI:** 10.1016/j.focus.2024.100282

**Published:** 2025-02-06

**Authors:** Mohammad Ehsanul Karim, Md Belal Hossain, Chuyi (Astra) Zheng

**Affiliations:** 1 School of Population and Public Health, The University of British Columbia, Vancouver, British Columbia, Canada; 2 Centre for Advancing Health Outcomes, Vancouver, British Columbia, Canada; 3 Department of Statistics, University of British Columbia, Vancouver, British Columbia, Canada

**Keywords:** Complex survey, effect modification, smoking initiation, mortality, survival analysis

## Abstract

**Introduction:**

The authors aim to examine the modifications of the relationship between early smoking initiation and overall mortality by race/ethnicity and sex using data from U.S. adults in the 1999–2018 National Health and Nutrition Examination Surveys.

**Methods:**

The authors analyzed data from 10 aggregated National Health and Nutrition Examination Surveys cycles focusing on noninstitutionalized U.S. civilian population aged 20–79 years. Smoking initiation was categorized by age, and the study's primary outcome was all-cause mortality. The authors employed Kaplan–Meier curves and Cox proportional hazards models to investigate the associations and adjusted for race/ethnicity, sex, and survey cycle. The authors also explored effect modification by race/ethnicity and sex.

**Results:**

The analysis included 50,549 participants. The authors found that early smoking initiation was significantly associated with increased all-cause mortality across all age categories, with higher hazard ratios for earlier initiation ages. The relationship exhibited variations by race/ethnicity and sex. Non-Hispanic White population showing the highest risk, followed by non-Hispanic Black subpopulation, but race/ethnicity interaction terms was not significant. Significant interaction by sex was observed.

**Conclusions:**

Early smoking initiation is significantly associated with increased risk of mortality, with noticeable differences by race/ethnicity and sex. These findings emphasize the need for prevention measures that are sensitive to demographic variations.

## HIGHLIGHTS

• This study utilized 2 decades of National Health and Nutrition Examination Surveys data from U.S., enhancing the study's robustness.
• The study adjusted survival analysis and moderated by key demographic variables.
• It found significant sex-based mortality differences linked to age of smoking.
• It found varying risks by race/ethnicity, despite nonsignificant interaction terms.
• There is need for tailored public health interventions based on demographic specifics.

## INTRODUCTION

Cigarette smoking continues to be one of the leading preventable causes of premature death both globally and in the U.S.^1^^,^^2^ Although the global prevalence of cigarette smoking has seen a decline, the total number of smokers worldwide has increased owing to population growth.^3^, ^4^, ^5^ In contrast, in the U.S., not only has the prevalence of cigarette smoking decreased, but the overall use of tobacco products has also declined. Despite these declines, smoking still accounts for over 480,000 deaths annually.^6^

The majority of cigarette smokers initiate the habit during their childhood and adolescence, often influenced by various cigarette and tobacco product marketing campaigns. Young smokers who indulge regularly are more susceptible to nicotine addiction and tend to remain exposed for longer durations, leading to extensive physical harm in the long run.^7^

Sex and race/ethnicity are critical considerations for effect modifications in the analysis of the relationship between early smoking initiation and mortality. These factors significantly shape individual smoking behaviors, exposure patterns, and responses to the health effects of smoking.^8^, ^9^, ^10^, ^11^ For instance, biological differences in nicotine metabolism, which vary across sexes and racial groups, can influence addiction potential and cessation success rates, thereby affecting health outcomes associated with smoking.^12^ Cultural norms and socioeconomic conditions, often intertwined with race and ethnicity, dictate smoking initiation ages and patterns. These factors impact access to cessation resources and are linked to different smoking-related health outcomes.^13^ Moreover, disparities in healthcare access related to sex and race/ethnicity may influence the observed associations between smoking initiation and mortality differently across various demographic groups.^14^ This highlights the need for tailored public health interventions that address specific needs and risks within different populations, enhancing the precision and relevance of this analysis for informing targeted health policies and interventions.

This study has 2 main objectives: (1) to re-examine the relationship between the age at which cigarette smoking begins and overall mortality and (2) to examine the modifications of this effect by race/ethnicity and sex, using data from U.S. adults in the 1999–2018 National Health and Nutrition Examination Surveys (NHANES) (spanning 10 cycles).

## METHODS

### Study Sample

The NHANES is a survey that is nationally representative, designed to collect information representative of the noninstitutionalized U.S. civilian population.^15^ It is conducted by the National Center for Health Statistics, a part of the Centers for Disease Control and Prevention. The survey employs a multistage, stratified cluster sampling design. For this study, the authors utilized data from 10 aggregated NHANES cycles spanning from 1999–2000 to 2017–2018. The 2019–2020 cycle remained incomplete owing to the coronavirus disease 2019 (COVID-19) pandemic. Consequently, the available data from this period are not conducive to population-based inferences. This recent cycle was therefore omitted from this study.

Data collection was primarily done through interviews. Mortality details were provided by the National Center for Health Statistics through linkage with public-use linked mortality files based on death records.^16^ In this analysis, the authors included adults aged between 20 and 79 years. Given that individuals aged ≥80 years are coded as 80 in the recent NHANES cycles, the authors excluded participants who were aged either below 20 or above 79 years as well as those with incomplete data on smoking status or mortality. Some records were considered ineligible for record linkage when they did not meet the minimum data requirements for mortality follow-up, such as when the identifying data were insufficient to conduct the linkage. These records, not available in the public release data, were considered incomplete.^16^, ^17^, ^18^

### Measures

NHANES has a question about age at which the study participant started smoking cigarettes regularly (SMD030). Exposure to regular smoking based on the age of initiation was categorized into the following groups: aged <10, 10–14, 15–17, 18–20, and >20 years. These categorizations have been used in previous research.^19^^,^^20^ The age groups for smoking initiation were selected to capture distinct developmental and addiction risk profiles, with the under 10 category included despite its small sample size to highlight the severe risks of very early smoking initiation for targeted prevention and policy formulation.^21^ The reference category was comprised those who smoked <100 cigarettes in their lifetime (they never started smoking regularly). The authors refer to them as never smokers in the remaining parts of this paper, extracted from those who responded negatively to the question *Smoked at least 100 cigarettes in life* (SMQ020). The 100-cigarette criterion is a widely recognized threshold in tobacco research for distinguishing between nonsmokers and those with any level of regular smoking experience. This definition stems from a need to have a practical, albeit somewhat arbitrary, benchmark for epidemiologic surveys and studies on tobacco use and its health implications.^22^

The authors designated the time of birth as the baseline in this analysis to prevent differential baseline assignments for exposed and unexposed participants. The primary survival outcome variable was the time from birth to all-cause mortality (i.e., death due to any reason). The person-months of follow-up were calculated by combining (1) the age at the interview (RIDAGEYR) with (2) the time from the interview date to either the date of death or the end of the follow-up period, the latter of which was provided in the public-use linked mortality files (PERMTH_INT).^18^ Given that the age at the interview was recorded in years, not in months, some measurement error was expected while calculating the survival outcome. Unlike many cross-sectional studies, this research was immune to reverse causality, given that the outcome (i.e., death) occurred after exposure to smoking.

Other variables of interest include the following categorical variables: sex (male, female), race/ethnicity (non-Hispanic White, non-Hispanic Black, Hispanic, and others), and survey cycle (1–10). Starting from the 2011–2012 cycle, NHANES introduced a new race/ethnicity category labeled non-Hispanic Asian. Adjusting for the survey cycle indicator provides a strategy to control for the myriad changes and variations, such as shifts in population characteristics, fluctuations in outcome prevalence, and potential variability in recruitment strategies, which can occur over time. This ensures that the derived associations remain robust and reduces the influence of potential temporal confounders. Appendix D (available online) provides the rationale of not adjusting for other covariates.

NHANES survey includes family poverty income ratio (PIR) (comparing household income with the poverty threshold adjusted for household size and composition, INDFMPIR) and the education level of the household head (DMDHREDU), which the authors have used in this sensitivity analyses as indicators or proxies of family SES.^23^ PIR is divided into the following 6 categories: 0–0.99, 1–1.99, 2–2.99, 3–3.99, 4–4.99, and >5.^24^ These variables were not included in the main analysis because (1) the information related to the variables may have been reported by the participants themselves, not by their parents, and (2) these variables exhibited a high degree of missing data. The authors did not attempt to impute the values because they believed that they cannot build a reliable imputation model on the basis of the available covariates.

### Statistical Analysis

The design was created on the entire data using the design features: interview weights, clusters, and strata. Subsequently, the authors subset the design to focus on eligible patients to estimate variances using the Taylor series linearization method. All subsequent analyses were adjusted appropriately for these design features to account for the features of the survey design. The authors considered *p*<0.05 as statistically significant. All analyses were conducted using R, Version 4.2.2.^25^

Cohort characteristics were summarized using sample counts and weighted proportions. To investigate the association between exposure to regular smoking by age of initiation and time from birth to mortality, the authors employed the Kaplan–Meier curve. The authors tested differences in survival curves using a log-rank test and estimated hazard ratios (HRs) along with their associated 95% CIs from the crude Cox proportional hazards (PHs) model. For the adjusted Cox PH analysis, the authors refined the model to account for sex, race/ethnicity, and survey cycle. The very high chi-square *p*-values (close to 1) across all variables and in the global test for the adjusted model suggest that there is no evidence of time-dependent covariate effects, implying that the HRs remain constant over time and thus meet the PH assumption required by the Cox model.

To explore the potential effect modification by race/ethnicity in the association of interest, the authors constructed separate Cox PH models, introducing an interaction term between exposure and race/ethnicity and adjusting for both sex and survey cycle. For assessing effect modification by sex, the authors added an interaction term between exposure and sex, adjusting for race/ethnicity and survey cycle. For the effect modification analysis (e.g., sex is the effect modifier), the authors have an adjusted Cox PHs model (say adjusted by race and survey cycle, where an interaction between sex and smoking initiation age is present); the impacts of interacting variables, such as a particular sex category and a specific smoking initiation age, are computed by summing the log-HRs (i.e., coefficients from the adjusted Cox model) for these variable categories (e.g., male and smoking initiation age before 10) and their interaction effect. Then, this sum is exponentiated to calculate the corresponding HR. The associated CIs for the log-HRs are calculated using the Wald method. Recognizing that smoking behavior is modifiable, whereas race/ethnicity and birth sex are constants, the authors chose an effect modification analysis over an interaction analysis.

The authors also reported further assessments of interaction terms, as follows:

**Testing interaction terms.** The current software implementation prohibits testing interaction terms for survey-featured Cox PH models.^26^ As a workaround, the authors approximated the Cox PH model using a survey-featured modified Poisson regression, with death as the outcome and the natural logarithm of follow-up time as an offset term.^27^ The authors assessed the interaction terms (for race/ethnicity and sex independently) using the Rao–Scott test on the basis of the estimated log-likelihood ratio.^28^

**Improvement in model fit.** The authors calculated the Akaike information criterion (AIC) for both effect modification models and compared these with the AIC from the adjusted model.

**Additive interaction**. In line with reporting guidelines, the authors have reported the additive interactions.^29^ The authors determined the relative excess risk due to interaction (RERI) using log-HRs derived from Cox PH models.

Although the authors have decided not to adjust for the smoking duration in the main analysis (for reasons explained earlier), they have investigated the relationship between the smoking duration and age of smoking initiation on the basis of only those participants for whom this information was available. The authors have further stratified this association on the basis of age and sex separately. As a sensitivity analysis, the authors have adjusted for additional variables that could be considered as proxies for family SES, such as family PIR and education level of the household head. The authors conducted additional analyses of effect modification by race/ethnicity for the cycles spanning 2011–2018, where the Asian race/ethnicity category was included.

## RESULTS

The analytic dataset comprised a sample size of 50,549. In total, 275 observations (about 0.5% of the entire sample size) were discarded owing to having missing exposure or outcome. There were no missing values for the covariates considered in the main analysis.

**Figure 1:**
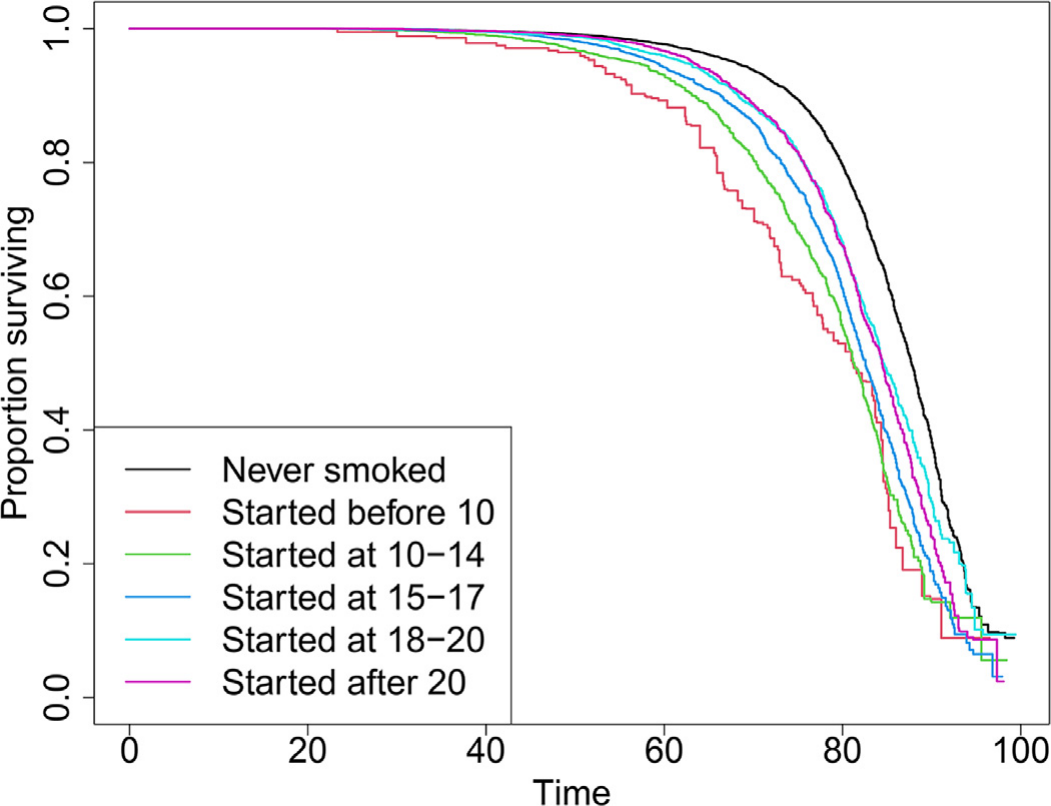
Survey-featured Kaplan–Meier curve (unadjusted) all-cause mortality within smokers initiating smoking at various ages and never smokers based on 1999–2018 National Health and Nutrition Examination Surveys.

Appendix Table 1 (available online) displays the summary statistics of the study participants, categorized by the outcome. The survey-featured chi-square tests associated with the exposure variable, race/ethnicity, sex, and survey year were all significant (*p*<0.001). Appendix Table 2 (available online) provides an additional table, stratified by exposure. The weighted Kaplan–Meier curve can be found in Figure 1. Each exposure level was significantly different from the never-smoker level (as determined by the z-statistic), and the chi-square statistic for the log-rank test was also significant.^12^^,^^25^

**Figure 2:**
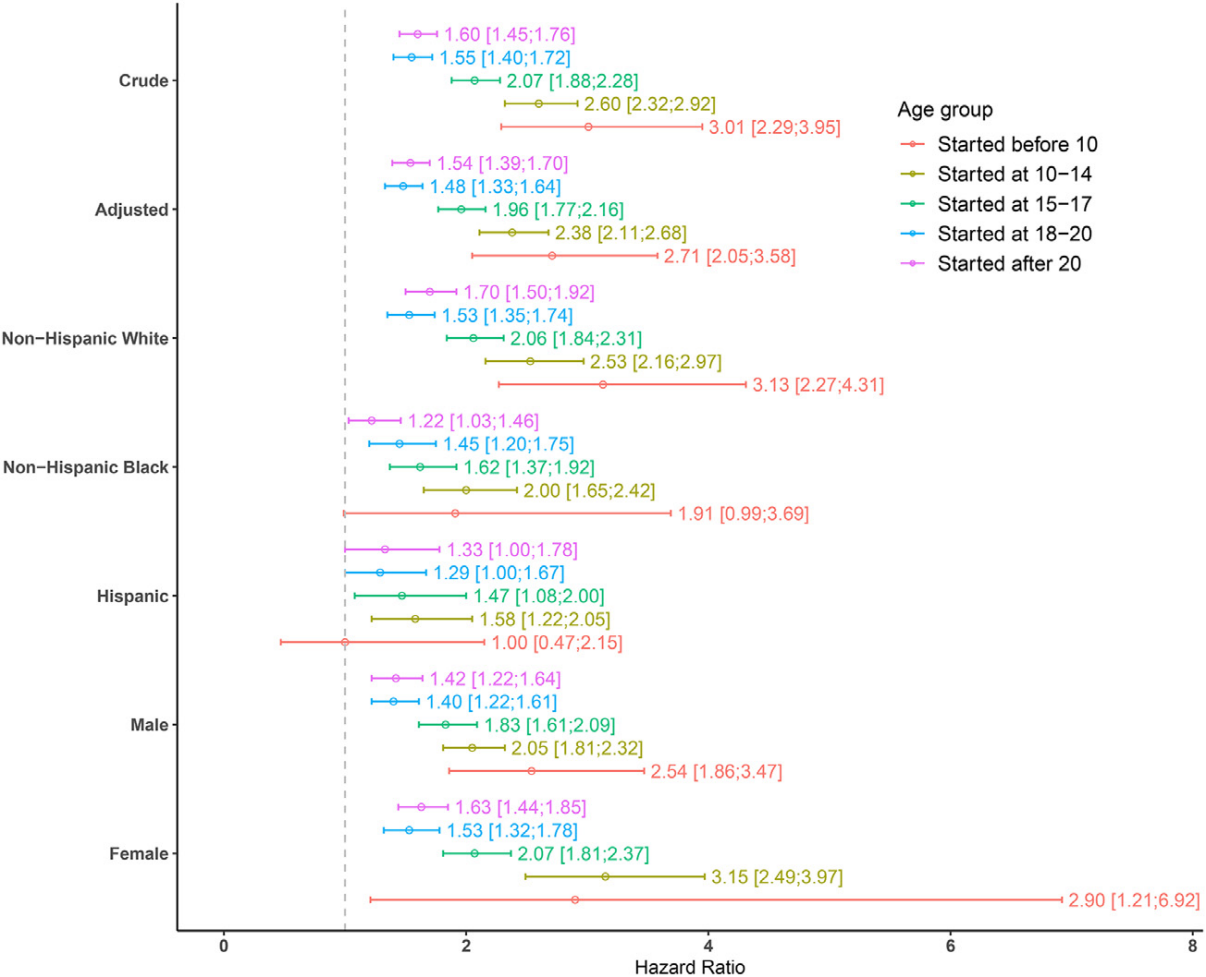
Estimates from crude and adjusted analyses (adjusted for sex, race/ethnicity, survey cycle indicator) and effect modification analysis for race/ethnicity and sex (adjusted for the remaining variables that are not considered effect modifier in the respective analysis) based on 1999–2018 National Health and Nutrition Examination Surveys. Dashed gray line indicates hazard ratio=1.

The authors observed a statistically significant association between exposure and all-cause mortality. In the unadjusted analysis, the risks associated with time to all-cause mortality were observed to increase gradually with earlier ages of smoking initiation. Specifically, the HR ranged from 1.6 to 3 for individuals who began regular smoking from age >20 years to those who started at ages <10 years. All associated *p*-values were significant (top of Figure 2). Upon adjusting for race/ethnicity, sex, and survey cycle indicators, the estimates decreased slightly (HR between 1.48 and 2.71), but the overall trend and significance remained consistent.

Regarding effect modification by race/ethnicity, the non-Hispanic White subpopulation exhibited the highest risk across all age-initiation categories, followed by the non-Hispanic Black subpopulation (middle part of Figure 2). Both of these groups demonstrated a similar trend, with the before age 10 years smoking initiation category showing the widest CIs owing to the limited sample sizes (Appendix Table 2, available online, provides the details), resulting in nonsignificant HR estimates for non-Hispanic Black and Hispanic groups initiating smoking before age 10 years.

The HRs for the female subpopulation were slightly higher than those of the male subpopulation (bottom part of Figure 2). Consistent with other analyses, the before age 10 years smoking-initiation category for females had the broadest 95% CI, given that only 80 females were categorized within that group (Appendix Table 2, available online, provides the details, and Appendix Table 3 shows a sensitivity analysis of these results as detailed below). However, all of the corresponding HR estimates related to males and females were significant.

Although the interaction term for race/ethnicity was not significant (*p*=0.16), the term for sex was significant (*p*=0.001) as per the Rao–Scott test. The model featuring a sex interaction (with the exposure variable) yielded a superior fit, producing an AIC of 17,858.35, in contrast to 17,872.62 from the adjusted model (which only accounted for main effects). However, the model with race/ethnicity interaction did not show any improvement, yielding an AIC of 17,878.17 when compared with the adjusted model.

RERI estimates are provided in Appendix Tables 4 and 5 (available online). According to the RERI estimates, additive interactions were statistically nonsignificant in the authors multiplicative Cox PH models for either race/ethnicity or sex. Therefore, there was no evidence of additive interaction.

Appendix Figure 1 (available online) reports the trends observed in the association between smoking duration and age of smoking initiation from the 1999–2016 surveys because information related to age of cessation was not collected in the 2017–2018 cycle. The graph is based on data from 8,916 participants who responded to questions related to ages of smoking initiation and cessation.

The trend in medians of smoking duration by smoking initiation age suggests that the median smoking duration are higher for those who initiates smoking earlier. This trend is also visible when the authors stratify the graphs by sex (Appendix Figure 2, available online) and race/ethnicity (Appendix Figure 3, available online).

Family PIR and education level of the household head were considered as proxies related to family SES. However, these variables were associated with high number of missing values, and consequently, 8,878 participants were removed from this sensitivity analysis. The results are reported in Appendix Figure 5 (available online). The HR values and trends are very similar to the ones reported for the main analysis (Figure 2). However, given smaller sample sizes, more HR estimates were found to be insignificant (Appendix Figure 5, available online).

In the survey cycles from 2011 to 2018, the authors observed that a majority of the Asian population fell into the never-smoker category. The sample sizes within each exposure category were relatively small, ranging from 5 to 213 (Appendix Table 3, available online, provides the details). For the Asian subpopulation, the highest HR was 1.53 (95% CI=0.43, 5.42) for those who began smoking between the ages of 10 and 14 years. Among all age categories for smoking initiation, none of the HRs were significant for the Asian subpopulation, with the exception of those who began smoking after the age of 20 years, which had an HR of 0.22 (95% CI=0.05, 0.93) (Appendix Figure 4, available online).

## DISCUSSION

In this study, the authors analyzed data from 10 cycles of NHANES to investigate the association between early smoking initiation and an incrementally increased risk of mortality. The Kaplan–Meier curve delineates a discernible dose–response relationship, with the association being significant across all age categories for smoking initiation. Even after adjusting for race/ethnicity, sex, and survey cycle indicators, the associations persisted, with HRs ranging from 2.71 (for those initiating before the age of 10 years) to 1.53 (for those initiating after the age of 20 years), in comparison with those among the never smokers. Given the higher risks associated with early smoking initiation observed in the results, the recent declining trends in smoking over the past decades in the U.S. appear to be particularly beneficial.

The interaction term for race/ethnicity was not significant at the 5% level, but the HRs within each subpopulation do show a pattern. A dose–response relationship remained evident within each racial category. The non-Hispanic White subpopulation exhibited the highest risk of all-cause mortality, followed by the non-Hispanic Black and then the Hispanic subpopulations. As described earlier, biological differences in nicotine metabolism, cultural norms, socioeconomic conditions, and disparities in healthcare access related to race/ethnicity may explain some of these trends.

In the analysis of the 4 most recent survey cycles, where the Asian category was incorporated, the authors observe a protective association between smoking initiation after the age of 20 years and mortality among the Asian subpopulation. The authors also observed that the majority of the Asian population fell into the never-smoker category. This suggests a lower prevalence of early smoking initiation within the Asian subgroup than within the other racial groups within the study cohort. However, this observation was based on relatively smaller sample sizes. However, for those who began smoking after the age of 20 years, the authors observed a statistically significant HR, despite the small sample size. This may suggest a distinct risk profile for late smoking initiators within the Asian population.

Effect modification by sex resulted in slightly higher HR estimates for the female subpopulation than for the male subpopulation. The corresponding interaction term for sex was significant. RERI analysis revealed no evidence of additive interaction for either term, partly owing to the wide CIs associated with them.

Various studies from different parts of the world have indicated an increased risk of mortality among individuals who began regular smoking early in childhood.^30^, ^31^, ^32^ Specific studies have also targeted males,^33^ females,^34^ and various racial groups.^9^

Although early smoking initiation is undeniably detrimental across all populations, the nuanced differences observed among late initiators in this study, particularly within the Asian subgroup, highlight the importance of culturally and demographically sensitive prevention strategies. Similar to this study, a previous study focusing on Chinese American adolescents also demonstrated a pattern of smoking onset different from that of White adolescents, with a generally later age of initiation.^35^

Two recent studies offer U.S.-based evidence highlighting elevated mortality rates among those exposed to smoking at an early age.^20^^,^^36^ The first study, based on National Health Interview Survey data, included participants recruited between 1997 and 2005 (with the National Health Interview Survey interview date serving as the baseline) and followed them for all-cause mortality until the end of 2011.^36^ This research further segmented the population into current and former smokers, finding HRs ranging from 0.97 to 1.24 for current smokers and between 1 and 1.19 when compared with HRs among those who commenced smoking at or after the age of 21 years. The second study enrolled participants from 1997 to 2014 and tracked them for cardiovascular mortality until the end of 2015. Using nonsmokers as a reference group and focusing solely on current smokers, this research revealed more pronounced HRs for cardiovascular mortality: from 4.89 (for those initiating smoking before age 10 years) to 2.25 (for those starting after age 20 years). Although these studies differed in their outcomes, the exposure categorization in the second study mirrored ours.

### Limitations

Because the authors have accounted for the complex survey design features of NHANES, their results hold national representativeness. The study also encompasses a longer follow-up duration than those of previous U.S. studies,^20^^,^^36^ allowing sufficient time for the evolution of long-term outcomes. However, the study did not account for cause-specific mortalities as potential outcomes. Owing to reliance on self-reported survey data, the results may be subject to bias.

Given that the authors did not adjust for all necessary confounders in the relationship of interest (e.g., family history, SES, and medical history) in the main analysis, caution needs to be applied when interpreting the results. The results are meant to be descriptive and not intended for causal interpretation. The authors conducted a sensitivity analysis with some proxies for SES variables, and the results did not differ noticeably. As explained earlier, NHANES does not collect adequate variables to regularly capture family medical history for the authors to adjust for them.

Smoking behaviors, such as duration and intensity, are important indicators for subsequent mortality and could be considered additional exposure variables. Below, the authors explain why that was not possible in the current project. First, variables such as duration can be calculated on the basis of self-reporting of smoking initiation and cessation ages (SMD030 and SMD055, respectively) as collected in the NHANES surveys. However, the collection of cessation age variables was discontinued in recent cycles of NHANES. The authors have conducted a sensitivity analysis with this variable in this work, but the sample size for this sensitivity analysis was drastically reduced because many participants (>80%) did not report both of these variables, which are necessary to calculate the smoking duration. In addition, participants may attempt to quit smoking several times during their lifetime, as reflected by SMQ670: Tried to quit smoking and SMQ848: number of times stopped smoking cigarettes, but the start-stop-restart dates were not captured in the data. Second, similarly, in the NHANES surveys, variables related to current smoking status (SMQ040) and recent smoking intensity (e.g., *In the last 30 days: how many days did you smoke a cigarette?* [SMD641] and *Average number of cigarettes per day* [SMD650]) are included. These variables capture intensity only on the basis of behavior during the past month of the survey, representing a single snapshot that may not adequately reflect lifetime smoking intensity, which can vary. Other information related to cessation, including the duration since quitting smoking cigarettes (SMQ050Q), age at which participants last smoked cigarettes regularly (SMD055), and attempts to quit smoking (SMQ670), is also collected. However, incorporating these into the analysis would require a complex analysis of time-dependent smoking exposures. In addition, these variables were subject to a high number of missing entries, potentially making the results of such a complex analysis unreliable. Therefore, although these concepts are useful, given data limitations within NHANES, the authors have not considered them in their analysis. Appendix E (available online) provides details for the future directions.

## CONCLUSIONS

Initiating smoking during childhood and adolescence significantly elevates the risk of premature and avoidable mortality, a finding corroborated by the authors analysis of the NHANES dataset spanning from 1999 to 2018, aligning with prevailing global data on the subject. The heightened risk, prevalent across varied racial/ethnic and sex groups (although not significant for race/ethnicity), underscores the imperative need for intensified public health initiatives focusing on curbing smoking initiation, particularly in the younger demographics. These revelations act as a stark reminder of the severe repercussions of early exposure to smoking and underline the urgent requirement for proactive preventive measures in an environment already fraught with multifarious health adversities. Governments and health organizations should, therefore, implement more rigorous strategies to dissuade smoking and advocate for cessation programs specifically targeting youth populations.

## Appendix Supplementary materials

Image, application 1

## Footnotes

Supplementary material associated with this article can be found in the online version at doi:10.1016/j.focus.2024.100282.
