# Supplementary material for: Examining the Role of Race/Ethnicity and Sex in Modifying the Association Between Early Smoking Initiation and Mortality: A 20-Year NHANES Analysis
Source: AJPM Focus. 2025 Feb 6;4(2):100282. doi: 10.1016/j.focus.2024.100282 (PMC12128641; doi:10.1016/j.focus.2024.100282)
Supplement: Supplementary file 1 [file mmc1.pdf]

Supplementary Content: "Examining the Role of Race/Ethnicity and Sex in Modifying the Association Between Early Smoking Initiation and Mortality: A 20-Year NHANES Analysis"

A Additional Summary Tables

Appendix Table 1: Characteristics of the study sample among US adults aged 20-79 years in the 1999–2000 to 2017–2018 NHANES, stratified by all-cause mortality.

Levels	Alive	Dead	Overall
	(N=44,377)	(N=6,172)	(N=50,549)
**Smoking**			
Never smoked	26,235 (57.99%)	2,358 (36.57%)	28,593 (56.04%)
Started before 10	244 (0.49%)	93 (1.48%)	337 (0.58%)
Started at 10-14	3,145 (7.17%)	758 (12.54%)	3,903 (7.66%)
Started at 15-17	6,003 (15.02%)	1,186 (21.01%)	7,189 (15.57%)
Started at 18-20	5,250 (12.24%)	1,004 (16.78%)	6,254 (12.65%)
Started after 20	3,500 (7.09%)	773 (11.61%)	4,273 (7.5%)
**Race**			
Non-Hispanic White	17,889 (66.42%)	3,180 (74.81%)	21,069 (67.18%)
Non-Hispanic Black	9,471 (11.35%)	1,506 (12.9%)	10,977 (11.5%)
Hispanic	12,342 (14.81%)	1,250 (7.87%)	13,592 (14.18%)
Others	4,675 (7.42%)	236 (4.42%)	4,911 (7.15%)
**Sex**			
Male	20,833 (47.88%)	3,558 (55.16%)	24,391 (48.54%)
Female	23,544 (52.12%)	2,614 (44.84%)	26,158 (51.46%)
**Survey year**			
1999-2000	3,188 (7.83%)	1,247 (19.75%)	4,435 (8.92%)
2001-2002	3,772 (8.42%)	1,073 (17.12%)	4,845 (9.21%)
2003-2004	3,583 (8.91%)	921 (15.36%)	4,504 (9.49%)
2005-2006	3,907 (9.46%)	667 (12.42%)	4,574 (9.73%)
2007-2008	4,705 (9.84%)	769 (10.81%)	5,474 (9.93%)
2009-2010	5,204 (10.28%)	563 (8.31%)	5,767 (10.1%)
2011-2012	4,770 (10.65%)	392 (6.73%)	5,162 (10.29%)
2013-2014	5,111 (11.11%)	285 (5.36%)	5,396 (10.59%)
2015-2016	5,132 (11.57%)	170 (2.79%)	5,302 (10.78%)
2017-2018	5,005 (11.92%)	85 (1.34%)	5,090 (10.96%)

NHANES: National Health and Nutrition Examination Survey.

[1] Frequencies are reported from the analytical sample.

[2] Percentages are calculated accounting for sampling (interview) weights.

Appendix Table 2: Characteristics of the study sample among US adults aged 20-79 years in the 1999–2000 to 2017–2018 NHANES (10 cycles), stratified by the exposure to regular smoking by age.

Levels	Never smoked	Started before 10	Started at 10-14	Started at 15-17	Started at 18-20	Started after 20	Overall
	(N=28,593)	(N=337)	(N=3,903)	(N=7,189)	(N=6,254)	(N=4,273)	(N=50,549)
**Race**							
Non-Hispanic White	10,194 (62.54%)	192 (74.05%)	2,115 (75.24%)	3,923 (76.89%)	3,019 (72.65%)	1,626 (63.69%)	21,069 (67.18%)
Non-Hispanic Black	6,334 (12.53%)	44 (6.92%)	669 (9.07%)	1,349 (8.47%)	1,356 (10.39%)	1,225 (15.77%)	10,977 (11.5%)
Hispanic	8,654 (16.5%)	73 (10.22%)	923 (12.37%)	1,511 (10.31%)	1,423 (11.09%)	1,008 (12.2%)	13,592 (14.18%)
Others	3,411 (8.42%)	28 (8.81%)	196 (4.32%)	406 (4.34%)	456 (5.87%)	414 (8.34%)	4,911 (7.15%)
**Sex**							
Male	11,534 (42.98%)	257 (73.85%)	2,495 (58.7%)	4,237 (56.37%)	3,661 (56.37%)	2,207 (50.42%)	24,391 (48.54%)
Female	17,059 (57.02%)	80 (26.15%)	1,408 (41.3%)	2,952 (43.63%)	2,593 (44.86%)	2,066 (49.58%)	26,158 (51.46%)

NHANES: National Health and Nutrition Examination Survey.
[1] Frequencies are reported from the analytical sample.
[2] Percentages are calculated accounting for sampling (interview) weights.

Appendix Table 3: Characteristics of the study sample among US adults aged 20-79 years in the 2011–2012 to 2017–2018 NHANES (4 cycles), stratified by the exposure to regular smoking by age.

Levels	Never smoked	Started before 10	Started at 10-14	Started at 15-17	Started at 18-20	Started after 20	Overall
	(N=12,371)	(N=126)	(N=1,495)	(N=2,762)	(N=2,434)	(N=1,762)	(N=20,950)
**Race**							
Non-Hispanic White	3,547 (59.51%)	71 (74.22%)	775 (73.86%)	1,314 (73.66%)	981 (68.27%)	551 (60.61%)	7,239 (63.82%)
Non-Hispanic Black	2,856 (12.39%)	14 (5.51%)	269 (7.49%)	575 (8.55%)	588 (11.03%)	568 (16.85%)	4,870 (11.59%)
Hispanic	3,387 (17.72%)	23 (11.12%)	328 (12.94%)	580 (11.46%)	540 (12.77%)	364 (12.87%)	5,222 (15.48%)
Asian	2,198 (7.62%)	5 (1.33%)	51 (1.31%)	171 (2.14%)	223 (3.4%)	199 (5.3%)	2,847 (5.65%)
Others	383 (2.76%)	13 (7.82%)	72 (4.4%)	122 (4.19%)	102 (4.53%)	80 (4.36%)	772 (3.45%)

NHANES: National Health and Nutrition Examination Survey.
[1] Frequencies are reported from the analytical sample.
[2] Percentages are calculated accounting for sampling (interview) weights.

B Smoking Duration

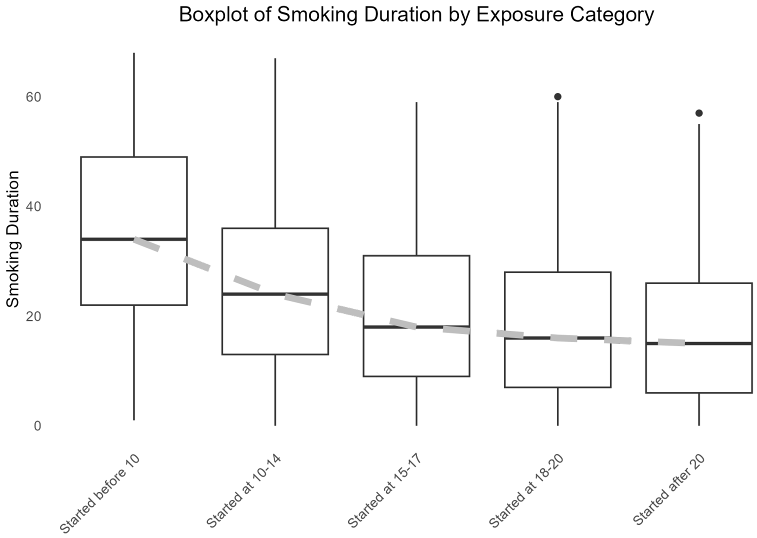

Appendix Figure 1: Trends in smoking duration based on age of smoking initiation from the 1999–2016 National Health and Nutrition Examination Surveys.

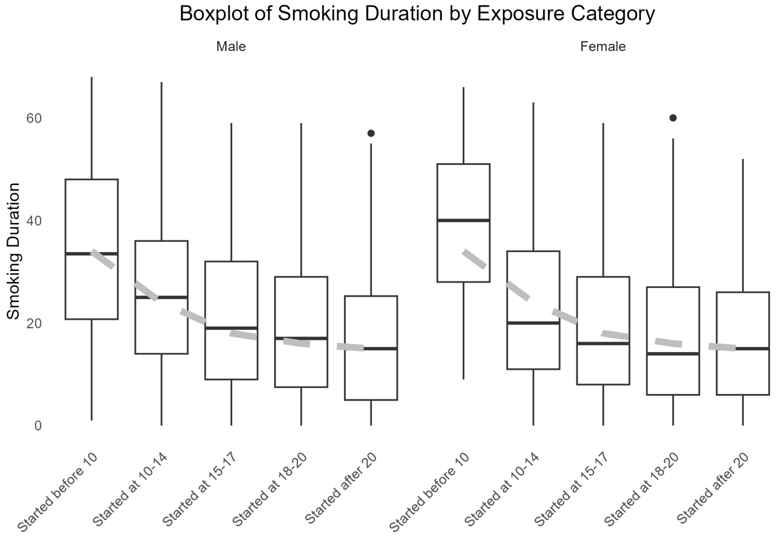

Appendix Figure 2: Trends in smoking duration based on age of smoking initiation, stratified by sex, from the 1999–2016 National Health and Nutrition Examination Surveys.

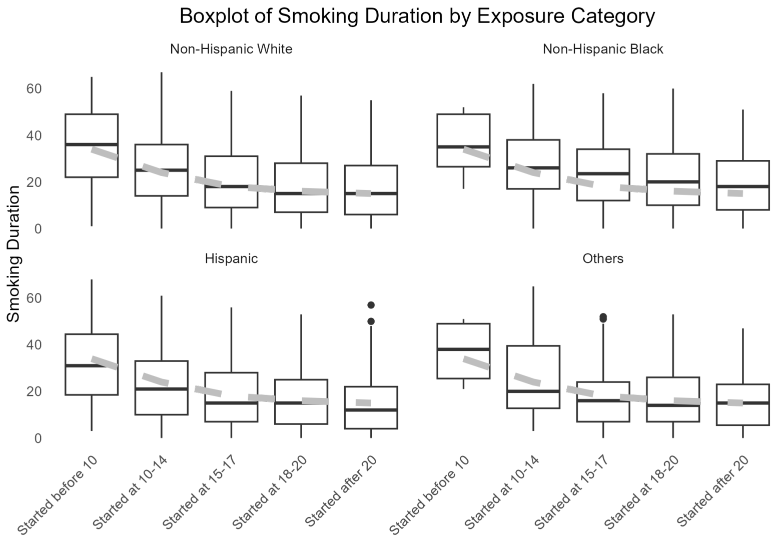

Appendix Figure 3: Trends in smoking duration based on age of smoking initiation, stratified by race/ethnicity, from the 1999–2016 National Health and Nutrition Examination Surveys.

C Additional Results from Effect Modification Analyses

C.1 Sensitivity analysis with 'Asian' category

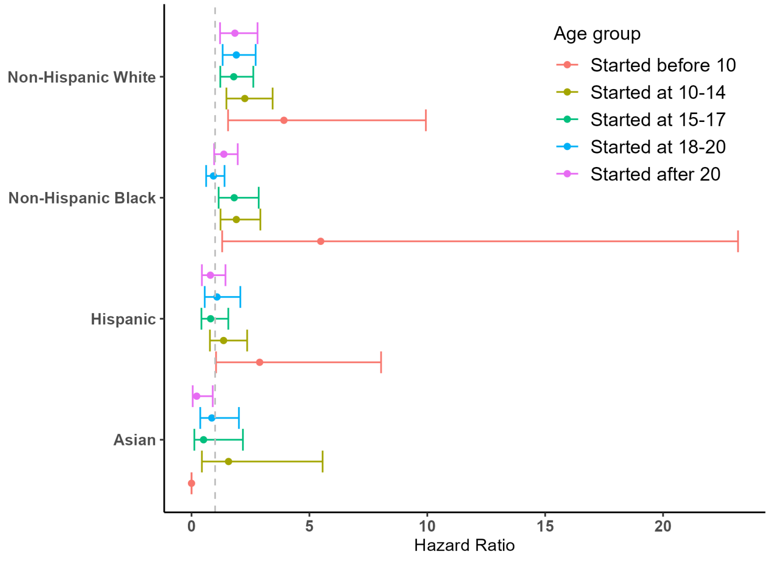

Appendix Figure 4: Estimates from effect modification analysis for race/ethnicity (adjusted for the race/ethnicity and survey cycle) based on 2011–2018 National Health and Nutrition Examination Surveys (NHANES). These surveys added an 'Asian' category. Confidence intervals are wider compared to the analysis utilizing 1999–2018 NHANES cycles due to lesser sample sizes. Dashed grey line indicates Hazard Ratio = 1.

C.2 Sensitivity analysis with proxy covariates

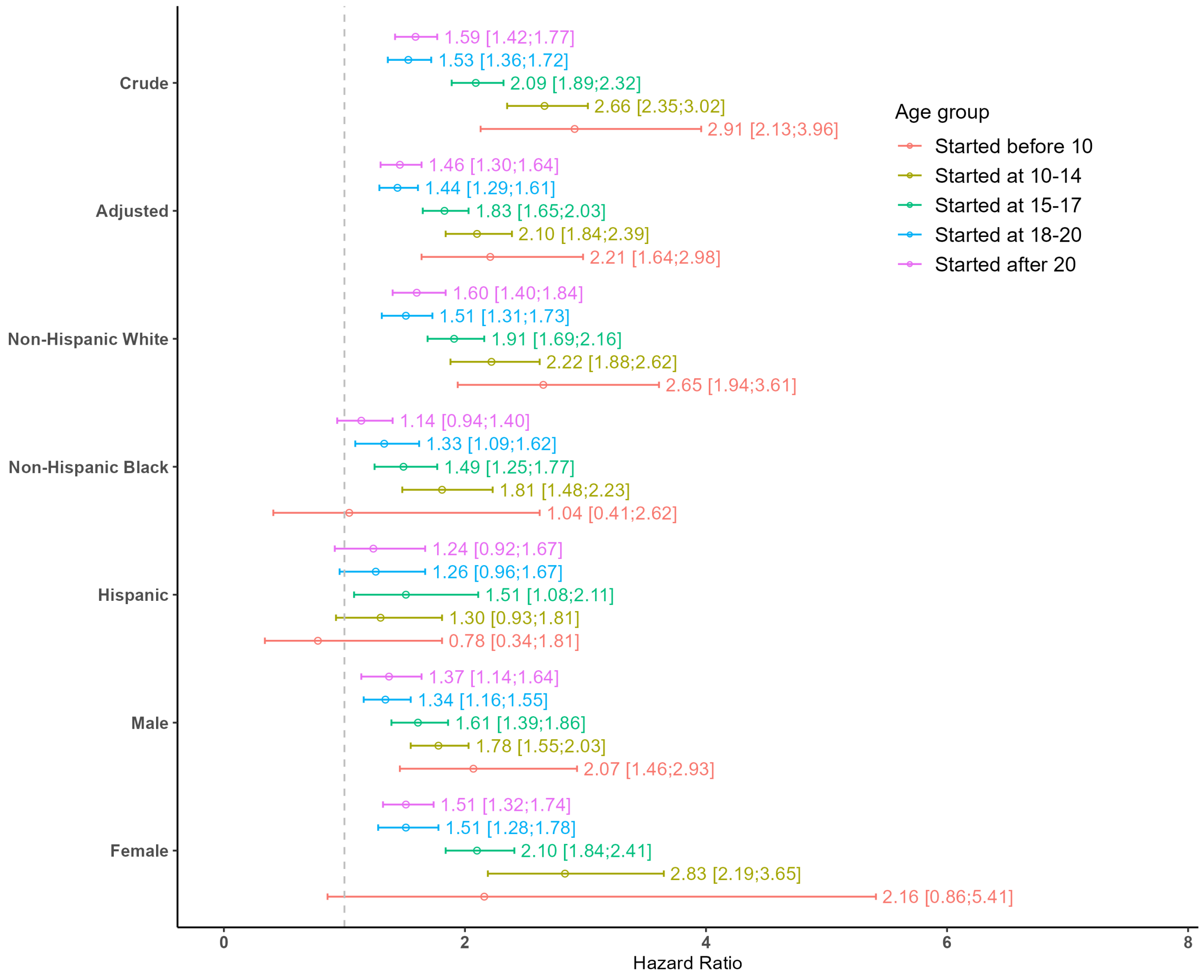

Appendix Figure 5: Estimates from crude and adjusted analyses (adjusted for sex, race/ethnicity, survey cycle indicator, family poverty income ratio and education level of the household head) and effect modification analysis for race/ethnicity and sex (adjusted for the remaining variables that are not considered effect modifier in the respective analysis) based on 1999–2018 National Health and Nutrition Examination Surveys. Complete case analyses results are reported based on 41,671 observations. Dashed grey line indicates Hazard Ratio = 1

C.3 Estimates from relative excess risk

Appendix Table 4: Estimates of RERI the model with exposure and race/ethnicity interaction.

Levels	RERI	95% CI
Started before 10 Non-Hispanic Black	-1.45	(-12.14,9.24)
Started at 10-14 Non-Hispanic Black	-0.79	(-6.58,5)
Started at 15-17 Non-Hispanic Black	-0.60	(-4.58,3.38)
Started at 18-20 Non-Hispanic Black	-0.20	(-2.67,2.27)
Started after 20 Non-Hispanic Black	-0.53	(-3.45,2.4)
Started before 10 Hispanic	-2.13	(-16.78,12.52)

Started at 10-14 Hispanic	-1.10	(-8.02,5.82)
Started at 15-17 Hispanic	-0.71	(-4.94,3.52)
Started at 18-20 Hispanic	-0.32	(-2.68,2.05)
Started after 20 Hispanic	-0.45	(-3.27,2.37)
Started before 10 Others	0.48	(-9.8,10.76)
Started at 10-14 Others	-0.04	(-5.88,5.81)
Started at 15-17 Others	0.18	(-5.17,5.53)
Started at 18-20 Others	-0.37	(-2.74,2)
Started after 20 Others	-0.64	(-3.78,2.5)

RERI: relative excess risk due to interaction; CI = confidence interval.

Appendix Table 5: Estimates of RERI the model with exposure and sex (Female) interaction.

Levels	RERI	95% CI
Started before 10	-0.26	(-5.8,5.27)
Started at 10-14	0.39	(-6.24,7.02)
Started at 15-17	-0.12	(-3.7,3.47)
Started at 18-20	-0.04	(-2.52,2.43)
Started after 20	0.01	(-2.69,2.71)

RERI: relative excess risk due to interaction; CI = confidence interval.

D Rationale of not adjusting for other covariates

Smoking behavior-related variables: As we are looking at the total effect of early initiation, including all its potential downstream consequences (e.g., continued smoking and its intensity), adjusting for these factors in our analysis may obscure the true relationship we are investigating, because these variables could act as mediators in the pathway from early initiation to mortality. We, therefore, diligently avoided adjusting for potential mediator variables. The same argument is true for other recorded variables related to smoking behavior (e.g., cigarettes per day, age of cessation, smoking history, use of other tobacco products, exposure to second-hand smoke).

Participant's demographic or behavioral information: Previous studies investigating related aims adjusted for additional factors [1, 2]. These include alcohol consumption and education of the participant (and physical activity, BMI, and income assistance are additionally considered in the first study), even though these were assessed much later than the early smoking initiation (e.g., often just prior to the survey data collection month). These factors, influenced by early smoking habits, can also sway the risk of mortality. Hence, they could serve as mediators in the relationship between early smoking initiation and mortality. Incorporating them into the analysis might obscure the genuine impact of early smoking on mortality, potentially skewing the results.

Family history: Additional variables such as physical health risk factors (e.g., genetic factors, family disease history) and family socio-economic status (educational status and income of the parents) would certainly help reduce confounding in our research question. We managed to adjust for the proxies of family socio-economic status in a sensitivity analysis.

Collection of information regarding family morbidity history in limited within NHANES. We could find family (or close relative) history of diabetes (`DIQ170`, `MCQ300c`), prediabetes (`DIQ175A`), heart attack (`MCQ300a`), asthma (`MCQ300b`) and prostate cancer (`SQ090D`). Since such information were collected sporadically or for only parts of the survey cycles we considered, we have not adjusted for these variables for consistency.

Geographical information: Geographic analysis using NHANES data is limited primarily because the survey does not provide detailed geo-location information, such as state or county identifiers, to the public. This limitation is in place to protect the privacy and confidentiality of survey respondents. While the `SDMVSTRA` variable is used for variance estimation and accounts for stratification in the survey design, it does not offer direct insights into

geographical variations across the United States [3]. The lack of detailed geographic data makes it challenging to conduct reliable and nuanced geo-related analyses or to draw region-specific conclusions. Therefore, while NHANES is a valuable resource for assessing health and nutritional statuses at a national level, its use for detailed geographic analysis is significantly constrained.

E Future Directions

Considering the broader implications of our findings, the primary focus of our study was to assess the relationship between the age of smoking initiation and all-cause mortality. We also acknowledge the potential for nuanced insights that could emerge from considering current smoking behavior. Empirical evidence suggests that heavier smoking patterns and sustained smoking into adulthood markedly exacerbate the risk of chronic diseases and premature death, highlighting the multifaceted nature of smoking behavior in health trajectories [4, 5]. Early initiators who modify their smoking behavior over time may exhibit different health outcomes compared to persistent heavy smokers, underscoring the complex interplay between initiation age and subsequent smoking behavior [6, 7]. Relevantly, previous analyses of NHANES data examined the implications of smoking on mortality, underscore the critical role of smoking behavior in health outcomes, albeit not directly focusing on smoking intensity [8, 9]. These considerations support the notion that a comprehensive evaluation of smoking behavior, encompassing initiation, intensity, and cessation, is vital for understanding and mitigating its health impacts. However, as mentioned above, the data collected by NHANES are not well-equipped to handle such analysis properly. Future research could explore these complex interactions and their implications for public health interventions when relevant variables are available.

Appendix References

[1] Choi SH, Stommel M. Impact of age at smoking initiation on smoking-related morbidity and all-cause mortality. American Journal of Preventive Medicine. 2017;53(1):33-41.

[2] Thomson B, Emberson J, Lacey B, Peto R, Woodward M, Lewington S. Childhood smoking, adult cessation, and cardiovascular mortality: prospective study of 390 000 US adults. Journal of the American Heart Association. 2020;9(21):e018431.

[3] National Center for Health Statistics. Variance Estimation Tutorial; 2024. Last accessed: 2024-04-06. https://wwwn.cdc.gov/nchs/nhanes/tutorials/varianceestimation.aspx.

[4] Strandberg AY, Strandberg TE, Pitkälä K, Salomaa VV, Tilvis RS, Miettinen TA. The effect of smoking in midlife on health-related quality of life in old age: a 26-year prospective study. Archives of internal medicine. 2008;168(18):1968-74.

[5] Jha P, Ramasundarahettige C, Landsman V, Rostron B, Thun M, Anderson RN, et al. 21st-century hazards of smoking and benefits of cessation in the United States. New England Journal of Medicine. 2013;368(4):341-50.

[6] Doll R, Peto R, Boreham J, Sutherland I. Mortality in relation to smoking: 50 years' observations on male British doctors. Bmj. 2004;328(7455):1519.

[7] Pirie K, Peto R, Reeves GK, Green J, Beral V. The 21st century hazards of smoking and benefits of stopping: a prospective study of one million women in the UK. The Lancet. 2013;381(9861):133-41.

[8] Borrell LN. The effects of smoking and physical inactivity on advancing mortality in US adults. Annals of Epidemiology. 2014;24(6):484-7.

[9] Jones MR, Tellez-Plaza M, Navas-Acien A. Smoking, menthol cigarettes and all-cause, cancer and cardiovascular mortality: evidence from the National Health and Nutrition Examination Survey (NHANES) and a meta-analysis. PloS one. 2013;8(10):e77941.
